# Effects of Lipids and Type of Amino Acid in Protein in Microalgae on Nitrogen Reaction Pathways during Hydrothermal Liquefaction

**DOI:** 10.3390/ijms241914967

**Published:** 2023-10-06

**Authors:** Tianyi Bao, Jesse Zhu, Nianze Zhang, Yuanyuan Shao

**Affiliations:** 1 School of Chemical Engineering and Technology, Tianjin University, Tianjin 300072, China; 2 Department of Chemical & Biochemical Engineering, The University of Western Ontario, London, ON N6A 3K7, Canada; 3 Zhejiang Institute of Tianjin University, Shaoxing 312300, China; 4 Zhejiang—Canada Joint Laboratory on Green Chemicals and Energy, Nottingham Ningbo China Beacons of Excellence Research and Innovation Institute, The University of Nottingham Ningbo China, Ningbo 315100, China

**Keywords:** amino acid, lipid, nitrogen, reaction mechanisms, pathway

## Abstract

It is meaningful to understand the conversion pathways of nitrogen during the hydrothermal liquefaction process of microalgae to reveal the related reaction mechanisms and develop effective methods to prevent N from ending in biocrude, which eventually increases the quality of biocrude. Extending from our previous works that mainly focused on two high-protein (>50 wt%) microalgae (*Chlorella* sp. and *Spirulina* sp.), *Nannochloropsis* sp., which has a high lipid content (>70 wt%), was used as the feedstock for this project using the same methodology. The high lipid content in *Na.* induced less nitrogen during the oil phase and as a result, reduced the heteroatom content while also improving the quality of biocrude. It is worth noting that another investigation was conducted on the model compounds with different types of amino acids to specify the effects of the types of amino acids in the proteins in microalgae on the N pathway and their distribution in the products (aqueous phase, oil, solid, and gas). It was found that the basic amino acid in microalgae caused the formation of more N-heterocyclic compounds in the biocrude. The mass flow based on the mass balance was demonstrated to further refine the map showing the predicted reaction pathway of nitrogen from the previous version.

## 1. Introduction

Given the climate change and environmental pollution dilemmas created by excessive utilization of fossil fuels, it is imperative to explore the liquid fuel production of microalgae via hydrothermal liquefaction (HTL) given their renewability, rapid growth rate [1], and reduced levels of energy consumption resulting from not needing to dry the raw feeds [2,3]. However, microalgae HTL’s ability to deal with nitrogen-containing organic compounds (NOCs) in biocrude has always been a concern, leading to some potential challenges including being at risk for undesirable emissions, a decrease in the quality of liquid oil, having tough refining processes, and possessing high downstream costs [4,5]. It is helpful to understand the conversion pathways of nitrogen during the hydrothermal liquefaction process of microalgae to reveal the related reaction mechanisms and develop effective methods to prevent N from ending up in the biocrude, which eventually increases the bio-oil’s quality.

Generally, the nitrogen components in microalgae are originally presented as proteins [6]. It was, thus, not surprising when the content of proteins in microalgae was high and lipids was low, as this suggested that NOCs’ concentration in biocrude was evidently high [7]. Our previous study focused on high-protein (>50 wt%) microalgae (*Chlorella* sp. and *Spirulina* sp.) [8]. For the two microalgae with very similar nitrogen concentrations, there was more N deposited in the biocrude from *Chlorella* sp. (*Ch.*) compared to *Spirulina* sp. (*Sp.*) under all operational conditions. It was thus speculated that the species of amino acids in the proteins played a crucial role on the N pathways during the hydrothermal conversion of microalgae. Gai et al. [9,10] reported that most NOCs tended to end up in an aqueous fraction during the HTL of *Chlorella pyrenoidosa* and *Spirulina platensis* due to the different types and quantities of amino acids in them. However, they did not mention the subcomponents, including amides and amines, diketopiperazines (DKPs), and N-heterocyclic compounds (NHCs). Other extensive studies [11,12,13] involving amino acids (i.e., alanine, glycine, valine, aspartic acid, etc.) were mainly targeted at the rection kinetics of amino acids as the model compounds of proteins. All literature explained the general hydrothermal reaction mechanism of amino acids as the simultaneous and competitive proceedings of decarboxylation and deamination. However, the N pathway during the microalgae HTL was not uniquely taken into account and is still less clear. Arginine (basic) and leucine (neutral) were identified as the dominant amino acids in *Ch.* and *Sp.*, respectively, which were employed in our previous works. Two model compounds of them were applied in the project’s HTL experiments.

On the other hand, the lipids of microalgae were also found to influence the N distribution to some extent. As reported, they could significantly determine the outcome of the formation processes of the NOCs in the biocrude, especially in the proceeding of amidation and the Maillard reaction [14]. Generally, the amidation reaction occurred between fatty acids and an amine group of amino acids or free ammonia, which were deaminated by amino acids. The Maillard reaction predominantly occurred between the amine group of amino acids and the carbonyl group in carbohydrates [15]. Therefore, the increase in lipid content in microalgae could hydrolyze more fatty acids during HTL, resulting in increased production of fatty acid amides during amidation. Meanwhile, the Maillard reaction was severely inhibited, mainly manifested with a significant decrease in the content of the NHCs in the biocrude. Therefore, the high lipid content in microalgae could lead to a fierce competition between the two reactions. The main reason behind this phenomenon may be related to the reaction between lipids and carbohydrates in the early stage of the HTL process [16]. The reaction could generate some carboxylic acids as intermediate products, resulting in an increase in the acidity of the reaction system. Koehler et al. [17] reported that the addition of acid could benefit from a decrease in the amount of NHCs in the biocrude since the increasing acidity of the system promoted the deamination of the amino acids. Moreover, Matayeva et al. [18] found that lipids exhibited a synergistic effect on the contribution of proteins into biocrude production through the extraction of certain hydrophilic NOCs, such as amines. This resulted in certain products, such as phenylethylamine, that could be transferred from the aqueous phase to the oil phase, thereby recovering the high-value-added products in the subsequent refinery and upgrading of the biocrude process. In addition, Yoo et al. [19] reported that the quality of biocrude was dependent on the lipids in microalgae. They found that the low reaction temperature during HTL was contributed to with the reduction in the nitrogen content in the biocrude, especially for the microalgae with a high lipid content. Therefore, the use of microalgae with a high lipid content as the feedstock was beneficial for the production of high-end biocrude.

*Nannochloropsis* sp. (*Na.*) is a type of lipid-rich microalgae and is usually considered to be one of the best biomass feedstocks for producing biodiesel through HTL. Its lipid content could reach over 68 wt% when dry, mainly consisting of C16 and C18 fatty acids [20]. In this project, *Na.* was included in the HTL experiments to further investigate how lipids influence the N distribution and pathway. Its inclusion also further deepens and broadens the analysis on the effects of different microalgae species under different reaction conditions based on our previous works [8].

## 2. Results

### 2.1. Type of Protein

**Figure 1:**
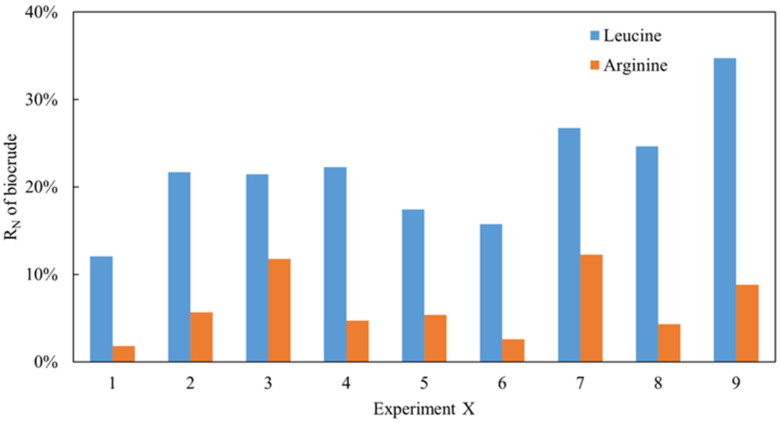
The RN of biocrude from HTL of leucine and arginine.

As reported in our previous works [8], the nitrogen recovery (R_N_) in biocrude obtained from *Ch.* was always higher than that of *Sp.*, which may be a result of the protein being composed of several different types of amino acid. As analyzed, leucine is a common protein in *Ch.* while arginine is dominant in *Sp.* Two model compounds with both amino acids were employed in the project to simulate the thermal conversion process using the same methodology. As shown in Figure 1, under all conditions, the R_N_ in the biocrude obtained from the arginine HTL was significantly lower than that of leucine despite arginine possessing a higher nitrogen content. Because of the low and similar levels of nitrogen content in solid residues detected, it can be inferred that the nitrogen in the raw material enters the aqueous phase typically when the N content is relatively low during the oil phase. This can also explain the distribution of nitrogen in the different phases of *Ch.* and *Sp.* with HTL and verify the aforementioned theories. Moreover, a range analysis of these orthogonal experiments of arginine and leucine was conducted as shown in Table 1 and Table 2, respectively. The K represents the sum of experimental data for a specific factor at a certain level. For example, K_1_, K_2_, and K_3_ in the first column represent the sum of the R_N_ values at T = 240, 260, and 280 °C, respectively. k is the average value of K. R is the range value, which is found by subtracting the minimum value from the maximum value of k in the same column. In general, the greater the R value, the greater the impact the factor has on R_N_. As shown in Table 1, 6.91 was the maximum value amongst the three R values. This suggests that the R_N_ of the biocrude from arginine was more sensitive to the solid loading rate. Similarly, the R_N_ of the biocrude from leucine (Table 2) was more sensitive to the temperature. Interestingly, the residence time was not a key factor for both model compounds. Additionally, the optimal reaction conditions for arginine were T = 260 °C, RT = 60 min, and SLR = 10%, while those for leucine were T = 240 °C, RT = 30 min, and SLR = 10% based on the smallest K values for each parameter, which represents the lowest R_N_ in the biocrude. A small solid loading rate could therefore be favorable for inducing less nitrogen into the oil phase during the HTL of amino acids.

**Figure 2:**
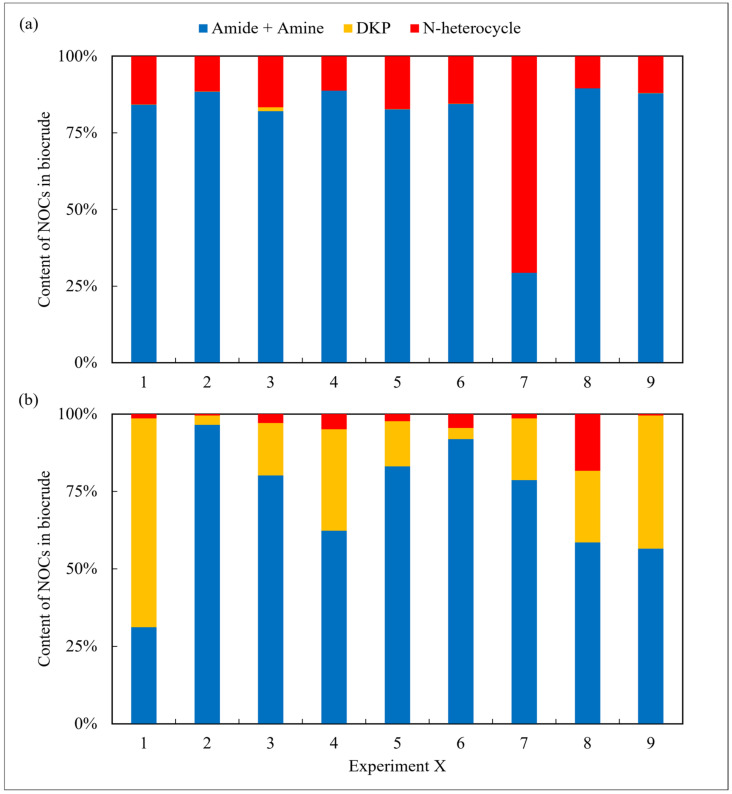
The forms of NOCs in the biocrude from HTL of two amino acids, respectively: (**a**) arginine and (**b**) leucine.

In addition, NOCs in the oil and aqueous phase were detected and analyzed using the GC-MS. Firstly, as shown in Figure 2, the derivatives of amides and amines were the main existing forms of nitrogen in the biocrude, with an average content of approx. 60–80% under most reaction conditions for both amino acids. However, the differences between them were the content of DKPs and NHCs. For arginine, the proportion of NHCs was high. On the contrary, the content of DKPs was significant for leucine. As commonly known [21], NHCs are more difficult to remove in the refining process compared to DKPs.

**Figure 3:**
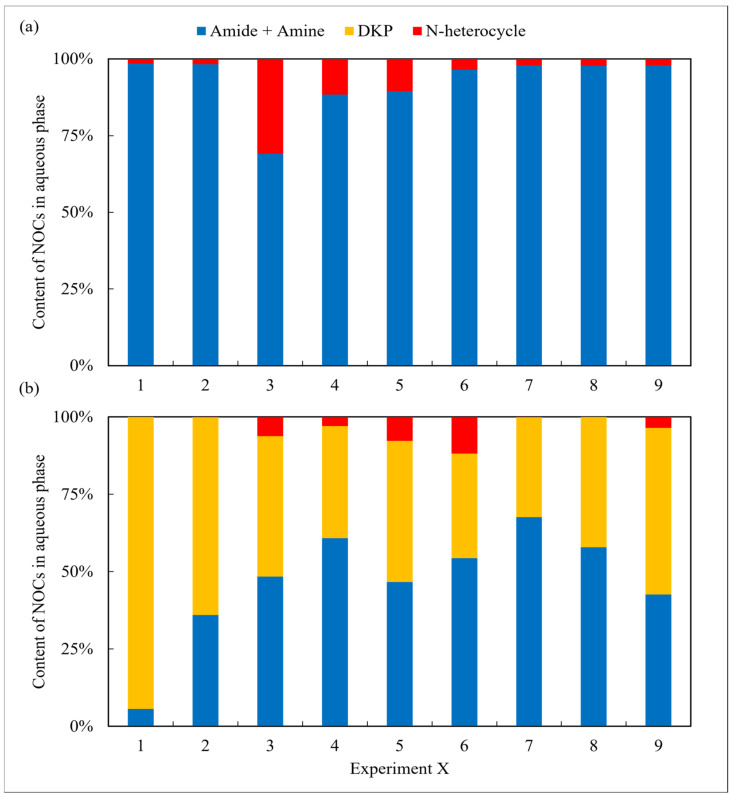
The forms of NOCs in the aqueous phase from HTL of two amino acids, respectively: (**a**) arginine and (**b**) leucine.

The aqueous phase obtained from arginine HTL contained high amounts of amides and amines in all cases and was nearly devoid of any DKPs (Figure 3). A similar result was also found in the oil phase. According to Hückel’s rule [22], the majority of the NHCs detected in the two phases in the case of the arginine possess aromaticity, whereas their counterparts in the aqueous phase from the leucine do not. The difference may be due to the assorted chemical structure of the two amino acids. Arginine contains strong alkaline guanidine groups, which undergo condensation, isomerization, and other reactions during HTL, making it easier to form more stable nitrogen-containing aromatic heterocyclic compounds [23]. Leucine has a relatively basic chemical structure and is more prone to intermolecular cyclize forming DKPs and its derivatives [24]. Overall, although the N contents in the oil phase from arginine are relatively low, further refining the NOCs is not easier than the counterparts obtained from leucine.

### 2.2. Temperature

**Figure 4:**
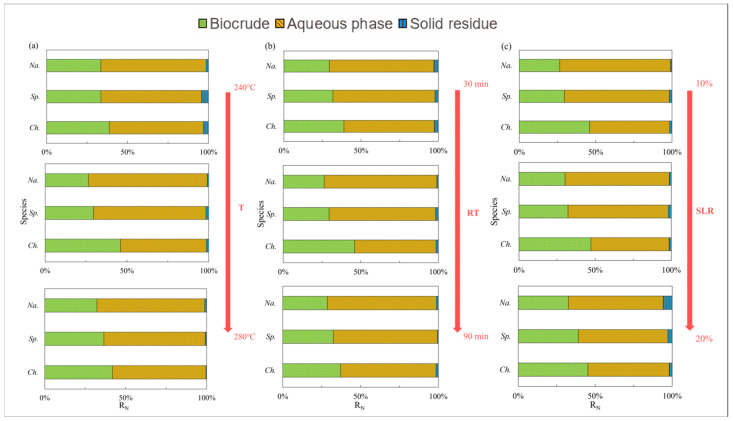
Nitrogen distribution of products from HTL of three microalgae under different conditions: (**a**) temperature (T); (**b**) residence time (RT); and (**c**) solid loading rate (SLR).

**Figure 5:**
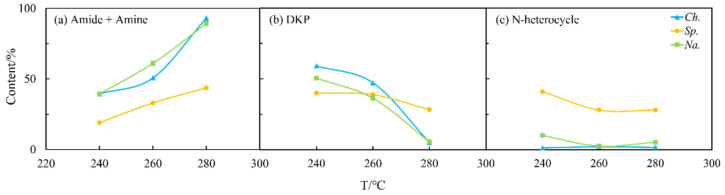
Effects of temperature (T) on the content of different NOCs: (**a**) Amide + Amine; (**b**) DKP; and (**c**) N-heterocycle in the biocrude from HTL of three microalgae.

**Figure 6:**
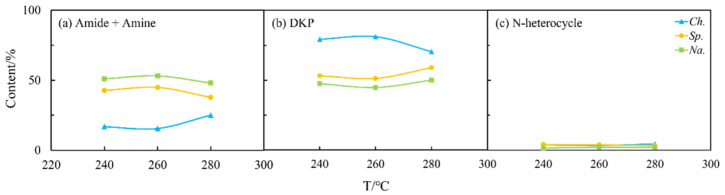
Effects of temperature (T) on the content of different NOCs: (**a**) Amide + Amine; (**b**) DKP; and (**c**) N-heterocycle in the aqueous phase from HTL of three microalgae.

The N distribution of nitrogen in the biocrude from the three selected microalgae under different HTL conditions is summarized and displayed in Figure 4, while Figure 5 presents the effects of temperature. For *Ch.*, 50 wt% of N was deposited in the biocrude at 260 °C (Figure 4a) when high amounts of amides and amines were detected (Figure 5a). As the temperature increased, the content of amides and amines gradually increased while DKPs (Figure 5b) decreased in the biocrude from *Ch.* and *Na*. This is consistent with findings in the literature [25], as many DKPs can be re-decomposed into free amino acids by changing the temperature, then decarboxylating to form amines, or deaminated into ammonia and finally, further acylating with fatty acids to form fatty amides. On the contrary, the existing forms of nitrogen for *Sp.* were less sensitive to the temperature either in the biocrude (Figure 5) or in the aqueous phase (Figure 6). This may be explained with the much more stable chemical structure of NOCs, which formed during Sp. HTL then decreased during the inter-conversions among different sorts of NOCs. As shown in Figure 6, although the contents of amides and amines and DKPs in the aqueous phase from *Ch.* were different from those of the other two microalgae at low temperatures, the content of NOCs from three microalgae gradually became more similar when the temperature was increased to 280 °C. This indicated that more complicated reactions took place at higher temperatures, producing more N-related intermediates generated at low temperatures to further transform and finally stabilize in the aqueous phase through a series of reactions.

### 2.3. Residence Time

**Figure 7:**
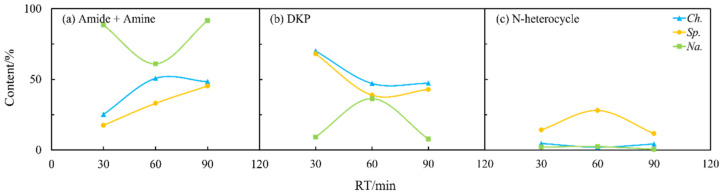
Effects of residence time (RT) on the content of different NOCs: (**a**) Amide + Amine; (**b**) DKP; and (**c**) N-heterocycle in the biocrude from the HTL of three microalgae.

**Figure 8:**
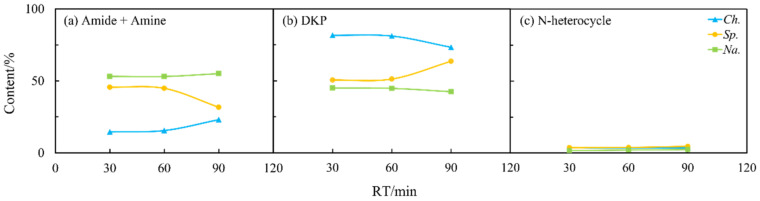
Effects of residence time (RT) on the content of different NOCs: (**a**) Amide + Amine; (**b**) DKP; and (**c**) N-heterocycle in the aqueous phase from HTL of three microalgae.

As shown in Figure 4b, the longer residence time could allow more nitrogen to be transferred into the aqueous phase and thus improve the quality of the biocrude as previously reported [26]. Figure 7 and Figure 8 illustrate that the contents of amides and amines, DKPs, and NHCs for *Sp.* in both the oil and aqueous phases varied drastically with residence time. The DKPs in the biocrude were eliminated while amides and amines and NHCs were increased from 30 to 60 min and kept stable. With the residence time extended to 90 min, the NHCs decreased significantly while amides and amines kept increasing. This suggests that some DKPs decomposed into amino acids, which participated in other reactions to form NHCs and amides at a short residence time. With the time extended, the chemical structure of DKPs became stabilized in both phases, where only some NHCs further reacted and transformed into amides and amines. For *Ch.* and *Na.*, the effects of the residence time on the formation of NHCs were less apparent compared to its effect on amides and amines and DKP. Amides and amines from *Na.* evidently decreased, initially from 30 to 60 min and then back to the initial content, which were directly opposite to those from *Ch.* It is worth noting that the increasing or decreasing of DKPs in the biocrude with residence time was always accompanied with the elimination or booming of amides and amines. This provides further evidence for the aforementioned inter-transformation between amides and DKPs.

Furthermore, as shown in Figure 8, there were less visible changes in the content of NOCs. This may indicate that the previously mentioned transformation reactions among NOCs mainly occurred during the oil phase. It may also indicate that most of the NOCs that remained in the aqueous phase were relatively stable regardless of changes in residence time [27].

### 2.4. Solid Loading Rate

**Figure 9:**
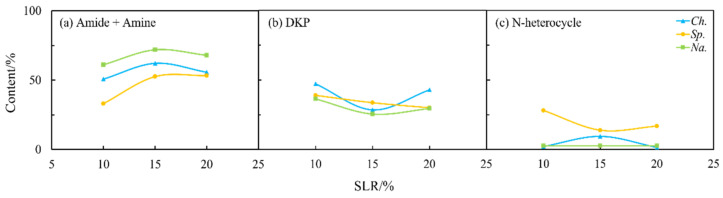
Effect of solid loading rate (SLR) on the content of different NOCs: (**a**) Amide + Amine; (**b**) DKP; and (**c**) N-heterocycle in the biocrude from HTL of three microalgae.

**Figure 10:**
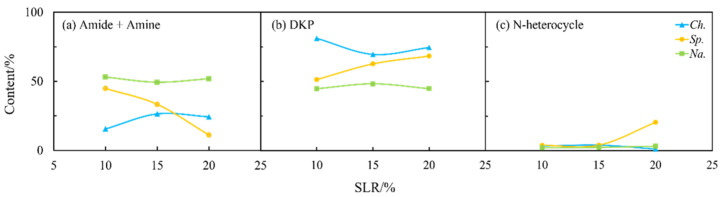
Effect of solid loading rate (SLR) on the content of different NOCs: (**a**) Amide + Amine; (**b**) DKP; and (**c**) N-heterocycle in the aqueous phase from HTL of three microalgae.

As shown in Figure 4 and Figure 9, the solid loading rate had some effects on the nitrogen distribution in the biocrudes and solid residues, especially for *Na.* and *Sp.* The content of amides and amines in the biocrude from three microalgae first increased and then more or less changed with the solid loading rate (Figure 9a) when DKPs were decreased (Figure 9b), which is consistent with the aforementioned theory on the transformation occurring between amides and amines and DKPs. Moreover, the solid loading rate had a significant effect on NOCs in both phases for *Sp.* (Figure 9 and Figure 10). The content of amides and amines gradually decreased with the solid loading rate while DKPs and NHCs kept increasing in the aqueous phase. The corresponding changes in the biocrude are directly opposite in particular for amides and amines and DKPs. These results demonstrated that the solid loading rate could not only change the distribution of nitrogen during HTL but also influence existing forms of nitrogen especially for *Sp.* [28]. However, the contents of NOCs in the biocrude and aqueous phase from *Na.* both changed very little. It can be inferred that *Na.* was not sensitive to the variations in the solid loading rate.

### 2.5. Microalgae Species

*Na.* with high lipid contents compared to *Ch.* and *Sp.* with high protein contents was used to further investigate the effects of microalgae species on the N pathway. As shown in Figure 4, nitrogen contents tended to remain in the aqueous phase as a witness to about a 60–70% portion compared to approx. 30–40% of NOCs presented in the form of oil-soluble compounds, when little nitrogen was found in the solid residue. Among the three applied species, the N distribution in the products from *Ch.* was most dependent on the reaction conditions. It can be inferred that the NOCs in the biocrude from *Ch.* were unstable, causing nitrogen to easily shift between the aqueous phase and oil phase [9]. Moreover, the nitrogen content in the biocrude from *Ch.* was higher than in the other two microalgae regardless of the operational conditions.

Not surprisingly, as shown in Figure 5c, Figure 7c andFigure 9c, the content of NHCs in the biocrude that converted from *Sp.* was evidently greater than that in the other two (*Ch.* and *Na.*). It is generally believed that NHCs are mainly formed via the Maillard reaction [15]. The results also demonstrated that reducing sugars obtained from the hydrolysis of carbohydrates in *Sp.* had a higher affinity for amines generated through decarboxylation from amino acids [29].

Moreover, for *Na.*—a species with high lipid contents—more nitrogen involved in the formation of amides and amines with lower molecular weights was distributed in the aqueous-phase product during HTL, while organic acids and their derivatives were dominant in the biocrude. Therefore, it can be concluded that the species of microalgae had a significant effect on the distribution and existing forms of nitrogen in the products of microalgae HTL.

## 3. Discussion

### 3.1. Mass Flow

**Figure 11:**
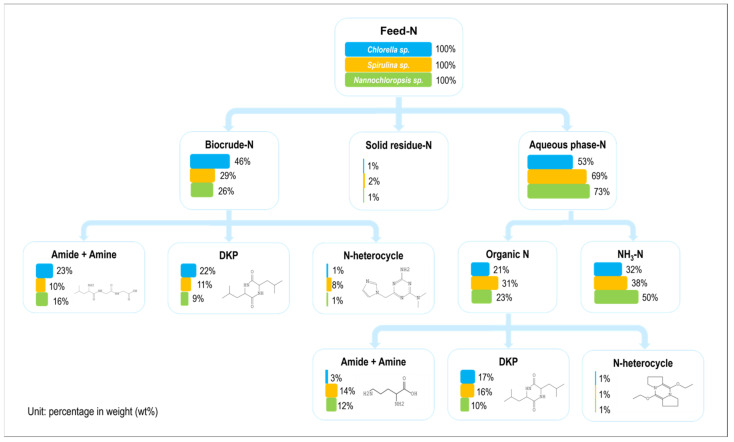
The mass flow of nitrogen during the HTL of three microalgae (condition: T = 260 °C, RT = 60 min, and SLR = 10 wt%).

Figure 11 illustrates the mass flow and existing forms of nitrogen during the HTL of three microalgae under the same reaction conditions. In general, the nitrogen in the microalgae prefers to end in the aqueous phase and primarily exists in the form of ammonia nitrogen [30,31]. Whether it was in the aqueous phase or biocrude, most of the organic nitrogen existed in the form of amides, amines, or DKPs, while a small amount existed in the form of NHCs like pyridine [32,33,34]. However, the proportion of NHCs in the biocrude from *Sp.* was evidently higher than that in the two other microalgae. Furthermore, the proportion of amides and amines was a bit lower, and the proportion of DKPs was close to the amides and amines whether it was in the aqueous phase or biocrude. It can be inferred that the intermolecular cyclization and the acylation reaction for amino acids are almost equally important during *Sp.* HTL [35]. The Maillard reaction also played a crucial role in the composition of the oil phase [36] as opposed to the results found for the other two microalgae. This further demonstrated that the similarity in the chemical compositions of microalgae cannot determine the nitrogen pathway during HTL [37,38]. Moreover, Figure 11 illustrates that nitrogen in the aqueous phase existed abundantly in the form of ammonium (approx. 70 wt%) for *Na.* The proportion of DKPs was relatively small whether it was in the biocrude or aqueous phase. This is because the content of DKPs, which was formed with the intermolecular cyclization of amino acids, was reduced during the initial stage of the reaction. It was replaced with the deamination of amino acids to form ammonia, which dissolved during the aqueous phase as ammonium nitrogen [39,40]. In contrast, the proportion of amides and amines in the aqueous phase was low in the case of *Ch.* Its nitrogen preferred to enter into the oil phase and exist in the NOCs except for NHCs.

### 3.2. Reaction Pathway

**Figure 12:**
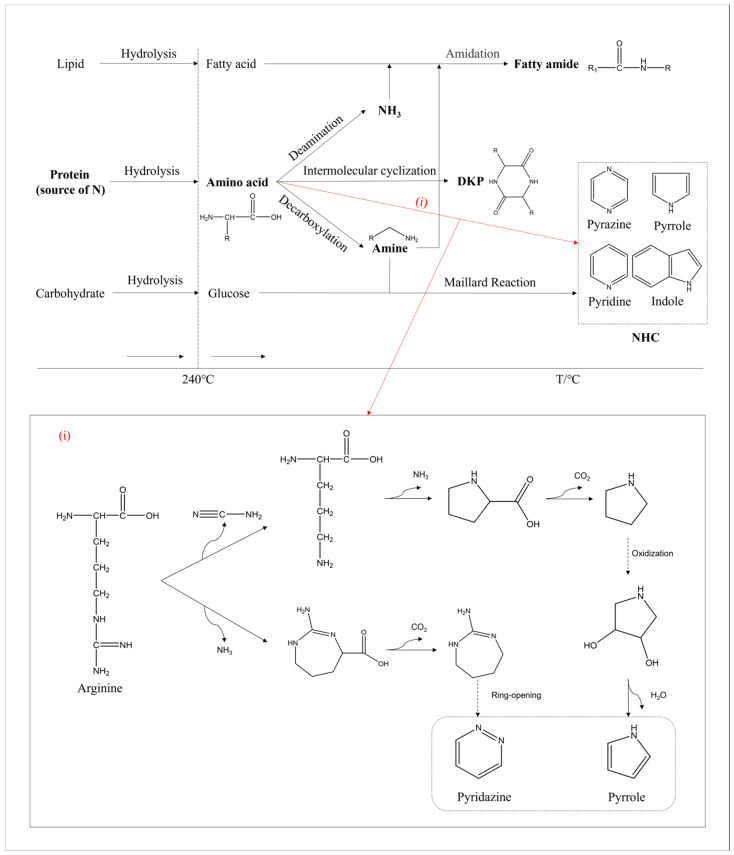
The reaction pathway of nitrogen during the HTL of microalgae. ((i) refers to the possible pathway from amino acids to NHCs).

As shown in Figure 12, a pathway map of nitrogen during HTL was further improved based on our previous study [8]. The hydrolysis of macromolecules occurred at a relatively low temperature. Lipids decomposed into glycerol and fatty acids, while proteins decomposed into amino acids and carbohydrates decomposed into monosaccharides such as glucose [41]. Upon increasing the temperature, some amino acids formed into ammonia through deamination, some into DKPs via the intermolecular formation of cyclization, and some into amines through decarboxylation [42,43]. Afterwards, the amidation reaction occurred when ammonia or amines reacted with fatty acids to form amides. Additionally, the Maillard reaction between amine and glucose produced NHCs such as pyridines and indoles.

In addition, a portion of the amino acids could be directly converted to NHCs as pathway (i) illustrates. Taking arginine as an example, one of the pathways we proposed was the removal of cyanide from arginine to form ornithine [44], which was an amino acid that was abundant during the aqueous phase as detected with the GC-MS. Afterwards, it would continue to undergo deamination and cyclization to form pyrrolidine [45]. Pyrrolidine was prone to oxidation at high temperatures, gradually forming the stable five-membered nitrogen-containing aromatic heterocycle called pyrrole [46]. The other pathway proposed was the deamination of arginine to form a seven-membered ring structure. As the reaction proceeded, it further decarboxylated and formed stable six-membered nitrogen-containing aromatic heterocycles such as pyrimidines through ring opening, rearrangement, and other reactions [47]. Moreover, some nitrogen-containing fused ring compounds could be formed with numerous and complex reactions.

## 4. Materials and Methods

### 4.1. Materials

China’s Shandong Jianchuan Biological Technology Co., Ltd. supplied three microalgae powders and two amino acid powders. Reagents were purchased from Yuanli Chemical Engineering Co., Ltd., Tianjin, China. The elemental and chemical compositions of the three microalgae are shown in Table 3. Compared with the other two microalgae, *Na.* has the highest oxygen content and the lowest nitrogen content. The lipid content of *Na.* is approximately twice that of the other two microalgae. In general, the chemical compositions of *Ch.* and *Sp.* are similar, while *Na.*, compared to the other two, possesses a higher lipid and lower protein content.

### 4.2. Methods

**Figure 13:**
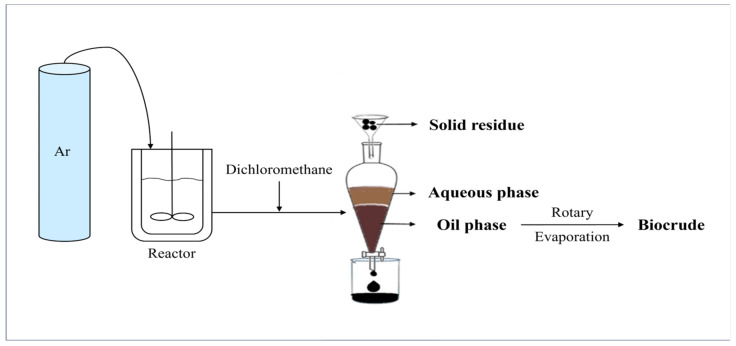
Schematic diagram of reaction and separation process.

The experimental procedure is shown in Figure 13. A 500 mL reactor was employed in this project. According to the calculations, the reactor was filled with a specific amount of microalgae powder and deionized water. Next, air in the reactor was purged using argon. To ensure that the reactor was well-sealed, a leak test was conducted after each injection of argon. The reactor was first heated using a furnace at a heating rate of 3 °C/min to a preset temperature. This was regarded as the starting point of the test, with the temperature remaining constant for the remainder of the preset residence time. The reaction was then stopped after shutting down the heater, and ice water was used to cool down the system quickly. Afterwards, the gas sample was collected using a sampling bag and then the remaining gas was completely discharged via the gas processing tube in the fume hood. After the reactor was opened, dichloromethane was added into the vessel twice to dissolve and wash out all products. After vacuum filtration, the filter residue, which was considered as the/used as the solid residue, was dried in a vacuum oven for 12 h. In order to separate the aqueous phase and oil phase, the filtrate was poured into a separatory funnel and allowed to sit for at least 30 min. In the separatory funnel, the aqueous phase was at the top and the organic phase was at the bottom. Finally, rotary evaporation at decompression conditions was employed to separate the biocrude from dichloromethane.

The separated gas and oil samples were collected and analyzed using the GC-MS (QP2010 ultra, Shimadzu, Kyoto, Japan) with a TR-WAXMS column and with a DB-5 column (30 m × 0.25 mm × 0.25 μm), respectively. The total N in aqueous samples was analyzed using testing kits (DR6000, HACH, Loveland, CO, USA) following HACH^®^ standard methods. Preliminary tests with three parallel repetitions were performed to determine the setting ranges for operational parameters including temperature, solid loading rate, and residence time. The tests also helped determine the experiment frequency to ensure data precision and enhance the test efficiency and performance by comparing the calculation of biocrude yield and nitrogen recovery (Equations (1) and (2)), gaseous and oil composition, as well as total nitrogen concentration in the aqueous phase. Consequently, two parallel tests were conducted for each preset run. Both biocrude yield and nitrogen recovery were calculated. When the standard deviation was less then 10% for both average values, one of the samples was randomly selected and sent for the above-mentioned qualification and quantitation analysis while another sample was stored in a freezer.

(1) $$Biocrude\;yield\;\left(wt\%\right)=\frac{Weight\;of\;biocrude}{Weight\;of\;microalgae}\times 100\%$$

(2) $$R_{N}\left(\%\right)=\frac{Biocrude\;yield\times content\;of\;N\;in\;biocrude}{Content\;of\;N\;in\;microalgae}\times 100\%$$

Due to the design of the controlled variable method used in the experiments of microalgae, which investigate the influence of each reaction condition on the characteristics of HTL products, the appropriate ranges of the conditions were obtained. A temperature of 240 to 280 °C, residence time of 30 to 90 min, and solid loading rate from 10 to 30% were employed in the HTL experiments of amino acids. In order to reduce the cost of time, the orthogonal experimental method was adopted to further explore the multiple effects of the three parameters on the quality of biocrude and aqueous phase from the two amino acids. Here, the data were analyzed using the range analysis to uncover the optimal combination of reaction conditions.

Additionally, a final chemical composition analysis of microalgae was also conducted following commonly used methods in the literature. The types of amino acids in *Ch.* and *Sp.* were detected and determined following the standard of GB5009.235-2016. All related analytical methods were presented in our previous publication [8] and other similar literature.

## 5. Conclusions

The differences between the amino acids composed of protein had significant effects on the diversity of the N distribution, which underpins our speculations. The high concentration of arginine (one basic amino acid) in *Spirulina* sp. promoted the formation of NHCs (very stable) in the oil phase, serving as the reason behind why nitrogen in the biocrude from *Spirulina* sp. could not be easily removed in the downstream even if its nitrogen recovery was lower than that of *Chlorella* sp. The high lipid content in *Nannochloropsis* sp. promoted greater nitrogen distribution in the aqueous phase, which could be reused for the cultivation of microalgae. Its distribution of organic nitrogen in the biocrude and aqueous phase was also relatively uniform with limited variations along with the reaction conditions. *Nannochloropsis* sp. is quite suitable for biofuel production as a bioresource.

Operational conditions have various and complex effects on the nitrogen reaction pathways depending on the species and compositions of microalgae due to the different reaction mechanisms. For the three applied microalgae, the nitrogen distribution showed different trends based on the temperature, but the proportion of amines and amides kept increasing in the biocrude, as some DKPs were decomposed into amino acids as the temperature further increased. These amino acids would react with the fatty acids and further decarboxylated or deaminated to produce fatty amides. Moreover, the residence time and solid loading rate also played a role. *Spirulina* sp. was more sensitive to the residence time and solid loading rate compared to the two other microalgae. In general, a longer residence time and lower solid loading rate could induce more nitrogen into the aqueous phase. However, the effects of this combination need to be investigated in greater depth and detail.

Lastly, the mass flow of nitrogen during HTL could help clarify and refine the reaction network of nitrogen pathways in a rigorous and systematic manner. Evidently, this is undesirable for the formation of NHCs. The Maillard reaction between the reducing sugar and amino acids and amino acid transformation are proposed as the primary formation mechanism, which requires more research and the use of advanced methodologies such as the density functional theory (DFT) calculation This will be a key topic in future research. In addition, it is worth further exploring the conversion mechanisms between various NOCs and other organic compounds, as well as the influence of other operational parameters such as catalysts or heating rates on the characteristics of HTL products. Reaction kinetics are also in need of more attention to achieve a better control of nitrogen content in products to meet different industrial needs.

## Figures and Tables

**Table 1:** The range analysis of the orthogonal experiment of arginine.

Experiment X	T/℃	RT/min	SLR/%	R_N_/%
1	240	30	10	1.80
2	240	60	20	5.67
3	240	90	30	11.75
4	260	30	20	4.72
5	260	60	30	5.38
6	260	90	10	2.59
7	280	30	30	12.27
8	280	60	10	4.29
9	280	90	20	8.83
Range analysis				
K_1_	19.22 (T = 240)	18.78 (RT =30)	8.68 (SLR = 10)	
K_2_	12.69 (T = 260)	15.34 (RT = 60)	19.21 (SLR = 20)	
K_3_	25.38 (T = 280)	23.17 (RT = 90)	29.40 (SLR = 30)	
k_1_	6.41	6.26	2.89	
k_2_	4.23	5.11	6.40	
k_3_	8.46	7.72	9.80	
R	4.23	2.61	6.91	

**Table 2:** The range analysis of the orthogonal experiment of leucine.

Experiment X	T/℃	RT/min	SLR/%	R_N_/%
1	240	30	10	12.08
2	240	60	20	21.71
3	240	90	30	21.46
4	260	30	20	22.27
5	260	60	30	17.44
6	260	90	10	15.75
7	280	30	30	26.75
8	280	60	10	24.63
9	280	90	20	34.75
Range analysis				
K_1_	55.24	61.10	52.45	
K_2_	55.46	63.78	78.73	
K_3_	86.13	71.95	65.64	
k_1_	18.41	20.37	17.48	
k_2_	18.49	21.26	26.24	
k_3_	28.71	23.98	21.88	
R	10.30	3.62	8.76	

**Table 3:** The Characteristics of three selected microalgae.

Microalgae	*Chlorella* sp. (*Ch.*)	*Spirulina* sp. (*Sp.*)	*Nannochloropsis* sp. (*Na.*)
Ultimate/wt%			
Carbon	47.13	45.82	43.41
Hydrogen	6.83	7.69	7.59
Nitrogen	10.31	10.41	6.96
Sulfur	0.64	0.61	0.64
Oxygen ^1^	35.09	35.47	41.40
Biochemical/wt%			
Protein	54.18	56.35	18.56
Lipid	38.46	35.79	71.63
Ash	0.75	0.89	1.13
Carbohydrates ^1^	6.61	6.97	8.68

^1^ Calculated with difference.

## Data Availability

Data are available on request from the corresponding author.
